# Effect of a Selective Mas Receptor Agonist in Cerebral Ischemia *In Vitro* and *In Vivo*

**DOI:** 10.1371/journal.pone.0142087

**Published:** 2015-11-05

**Authors:** Seyoung Lee, Megan A. Evans, Hannah X. Chu, Hyun Ah Kim, Robert E. Widdop, Grant R. Drummond, Christopher G. Sobey

**Affiliations:** 1 Vascular Biology and Immunopharmacology Group, Department of Pharmacology, Monash University, Clayton, Victoria, 3800, Australia; 2 Cardiovascular Disease Program, Biomedicine Discovery Institute, Monash University, Clayton, Victoria, 3800, Australia; 3 Department of Surgery, Southern Clinical School, Monash University, Clayton, Victoria, Australia; School of Pharmacy, Texas Tech University HSC, UNITED STATES

## Abstract

Functional modulation of the non-AT_1_R arm of the renin-angiotensin system, such as via AT_2_R activation, is known to improve stroke outcome. However, the relevance of the Mas receptor, which along with the AT_2_R forms the protective arm of the renin-angiotensin system, as a target in stroke is unclear. Here we tested the efficacy of a selective MasR agonist, AVE0991, in *in vitro* and *in vivo* models of ischemic stroke. Primary cortical neurons were cultured from E15-17 mouse embryos for 7–9 d, subjected to glucose deprivation for 24 h alone or with test drugs, and percentage cell death was determined using trypan blue exclusion assay. Additionally, adult male mice were subjected to 1 h middle cerebral artery occlusion and were administered either vehicle or AVE0991 (20 mg/kg i.p.) at the commencement of 23 h reperfusion. Some animals were also treated with the MasR antagonist, A779 (80 mg/kg i.p.) 1 h prior to surgery. Twenty-four h after MCAo, neurological deficits, locomotor activity and motor coordination were assessed *in vivo*, and infarct and edema volumes estimated from brain sections. Following glucose deprivation, application of AVE0991 (10^−8^ M to 10^−6^ M) reduced neuronal cell death by ~60% (P<0.05), an effect prevented by the MasR antagonist. By contrast, AVE0991 administration *in vivo* had no effect on functional or histological outcomes at 24 h following stroke. These findings indicate that the classical MasR agonist, AVE0991, can directly protect neurons from injury following glucose-deprivation. However, this effect does not translate into an improved outcome *in vivo* when administered systemically following stroke.

## Introduction

Stroke is a major neurovascular disease and a leading cause of mortality and long-term disability. While 15 million people worldwide suffer a stroke each year, the only pharmacological therapy available for stroke patients—the clot-busting agent, tissue plasminogen activator (t-PA)–must be administered strictly within a 4.5 h window from the time of stroke onset. As a result, less than 15% of stroke patients are eligible to receive t-PA and so there is a great need to identify additional therapies that may improve stroke outcome [1].

Accumulating evidence suggests that selective targeting of the renin-angiotensin system can provide neuroprotection during cerebrovascular diseases such as stroke. It is well established that excessive stimulation of angiotensin type 1 receptor (AT_1_R) by angiotensin II exerts detrimental effects in stroke. On the contrary, effects of the angiotensin type 2 receptor (AT_2_R) and the Mas receptor (MasR), which together form the protective arm of the renin-angiotensin system, are still relatively unexplored in stroke. We recently reported that the AT_2_R agonist, CGP42112, can directly protect neurons from ischemia-like injury *in vitro*, and that systemic administration of CGP42112 can reduce functional deficits and infarct volume following cerebral ischemia *in vivo* [2]. Moreover, previous studies have demonstrated that intracerebroventricular (ICV) pre- and post-treatment with CGP42112 to spontaneously hypertensive rats (SHR) reduced infarct volume, motor deficit and neuronal death, compared to the vehicle following the middle cerebral artery occlusion (MCAo) [3, 4]. Importantly, the effects of CGP42112 were confirmed to be AT_2_R-mediated as this protection was reversed in the presence of the AT_2_R antagonist, PD123319. Furthermore, recent studies have reported that pre- and/or post-treatment with the novel nonpeptide AT_2_R agonist, Compound 21 (C21), also reduces infarct volume and neurological deficits in rodent models of ischemic stroke [5–7]. These neuroprotective effects of C21 were attenuated by co-administration of PD123319, suggesting an AT_2_R-mediated effect.

The MasR has been identified as a functional receptor for angiotensin 1 to 7 [Ang (1–7)] [8]. Ang (1–7) exerts vasodilatory, antioxidative and anti-inflammatory effects through activation of the MasR, and this action has been well characterized in numerous tissues, including the brain where MasR expression is highest [9–12]. Previous studies have reported that central administration of Ang (1–7) results in reduced infarct volume and improved neurological outcome in a rat model of stroke [13–16]. However, Ang (1–7) is not selective for the MasR, as it also exhibits binding affinity for the AT_2_R [17–20]. Therefore, is unclear to what extent the protective effects of Ang (1–7) might have been mediated by the MasR, and it remains to be determined whether selective MasR activation represents a therapy that can independently elicit neuroprotection in cerebral ischemia. Currently, there is only one selective MasR agonist, AVE0991, which is a non-peptide based compound that lacks affinity for AT_2_R [20]. Thus, in the present study, we have assessed the efficacy of the selective MasR agonist, AVE0991, to provide neuroprotection under ischemia-like conditions *in vitro*, and following post-ischemic administration in an *in vivo* model of stroke.

## Materials and Methods

### Ethical Statement

This study followed the ARRIVE Guidelines and was conducted in accordance with the National Health and Medical Research Council of Australia guidelines for the care and use of animals in research, and with approval from the Monash University Animal Ethics Committee (Projects SOBSB/2014/064 and SOBS/2010/45). Under ethics committee guidance, we implemented and followed a monitoring protocol involving a clinical signs severity scoring system (approved by our ethics committee), where clinical signs (including activity, appearance, body weight, body temperature, breathing, coat, feces/urine and vocalization) were observed and assigned a score (i.e., 0, 1, 3 and 10) based on the animals’ condition per category. Animals with a total score of 0–4 were routinely monitored twice daily (at 9 am and 5 pm). Animals with a total score of 5–9 were more closely monitored—at least once every 3 hours. Animals with a total score of 10 or more were immediately euthanised.

### Animals

For the *in vitro* model of stroke, twelve pregnant FVB/N female mice from the Monash-bred colony were used to obtain cortical neurons from E15-17 pups of mixed sex. For the *in vivo* model of stroke, a total of 53 male C57BL6/J mice (mean = 8 weeks old, range = 6–10 weeks old; mean = 26 g, range = 22–30 g) from the Monash-bred colony were studied. The mice were maintained in the animal facility under specific pathogen free conditions on a 12 h light/dark cycle and had free access to water and food pellets before and after surgery. Five mice were excluded from the study because: (1) no measured increase in blood flow at reperfusion after 1 h ischemia (n = 3) and (2) death before 24 h of reperfusion had elapsed (n = 2).

### Primary Cultures

Cortical neuronal cultures were prepared, as previously described [2]. Briefly, time-mated pregnant mice were anesthetized using inhaled isoflurane and euthanized via decapitation. Embryos were immediately removed and placed into ice-cold Ca^2+^/Mg^2+^-free Hank’s balanced salt solution (HBSS; Invitrogen, Melbourne; supplemented with HEPES (20 mM; Invitrogen, Melbourne) and gentamicin (25 μg/ml; Invitrogen, Melbourne), then the cortices were carefully dissected out and freed from the meninges. To obtain a single cell suspension, cortices were digested in trypsin (1 mg/ml; Sigma, Sydney) for 10 min at room temperature, then neutralized with trypsin inhibitor (1 mg/ml; Sigma, Sydney). Dissociated cells were resuspended in Neurobasal medium (NBM; Invitrogen, Melbourne; pH 7.2) supplemented with B-27 (2%; Invitrogen, Melbourne), HEPES (20 mM), L-glutamine (1.3 mM; Invitrogen, Melbourne) and gentamycin (50 mg/ml; Invitrogen, Melbourne), then dispensed into poly-D-lysine (Sigma, Sydney) coated 60-mm^2^ Petri dishes (BD Biosciences, Franklin Lakes, USA). Cells were then incubated overnight in a humidified incubator (37°C; 5% CO_2_, Forma Scientific, Waltham, USA). Medium was replaced with fresh NBM (+ supplements), and the cells were maintained for a further 7–9 d with renewal of half of the medium every 3–4 d.

### Glucose Deprivation and Cell Viability Assay

For glucose deprivation, cultured primary neurons (7–9 d *in vitro*) were incubated in glucose-free Locke’s buffer (composition in mmol/L: NaCl 154.0, KCl 5.6, CaCl_2_2.H_2_O 2.3, MgCl_2_6.H_2_O 1.0, NaHCO_3_ 3.6, HEPES 5.0; pH 7.2) supplemented with gentamicin (25 μg/ml) for 24 h in a humidified incubator. Neurons received one of the following treatments: (i) control (no treatment); (ii) vehicle (Locke’s buffer only); (iii) AVE0991 (1x10^-8^, 1x10^-7^ or 1x10^-6^ M at the commencement of glucose deprivation (0 h) or 1x10^-7^ M at 0 h, 1 h, 4 h, or 8 h after glucose deprivation; MedChem Express; Monmouth Junction, NJ, USA); (iv) A779 (1x10^-6^ M; Auspep; Tullamarine, Australia); (v) AVE0991 (1x10^-8^, 1x10^-7^ or 1x10^-6^ M) in combination with A779 (1x10^-6^ M). Cell viability was determined using trypan blue exclusion assay. Following 24 h treatment, cells were incubated in 0.2% trypan blue (Sigma, Sydney) for 15 min in a humidified incubator. Cells were rinsed twice with pre-warmed phosphate-buffered saline (PBS; pH 7.2) then fixed with ice-cold 4% paraformaldehyde for 10 min at room temperature. Twenty images of random fields at 200x magnification were photographed in each dish using an inverted microscope and camera (E2000-U Nikon Eclipse; Nikon, Japan) and associated computer software (NIS-elements version 3.0; Nikon, Japan). Approximately one thousand cells were counted manually from each dish with the assistance of image analysis software (ImageJ; NIH, Bethesda, USA). In order to determine the amount of cell death naturally occurring during the experimental treatment period (i.e. independent of treatment), one neuronal culture dish was randomly chosen and a cell viability assay performed 1 d prior to the treatment. Percent cell death was the primary endpoint of this experiment. Investigators were blinded as to the treatment groups when obtaining images and counting cells.

### Cerebral Ischemia-Reperfusion

Focal cerebral ischemia-reperfusion was induced as described previously [2, 21]. Briefly, mice were anesthetized between 9.00 am and 1.00 pm with a mixture of ketamine (80 mg/kg i.p.) and xylazine (10 mg/kg i.p.). Rectal temperature was monitored and maintained at 37.0 ± 0.5°C throughout the procedure with a heat lamp, until animals regained consciousness. Following a midline neck incision, the right external carotid and pterygopalatine arteries were isolated and cauterized. The internal carotid artery was lifted and occluded at the peripheral site of its bifurcation as soon as the distal common carotid artery was clamped. Focal cerebral ischemia was induced by intraluminal filament occlusion of the right middle cerebral artery (MCA) for 1 h using a 6–0 nylon monofilament with a silicone-coated tip (0.20–0.22 mm, Doccol Co., Redlands, CA, USA). Severe (~80%) reduction in regional cerebral blood flow was confirmed using trans-cranial laser-Doppler flowmetry (PF5010 LDPM Unit, Perimed; Järfälla, Sweden) in the area of cerebral cortex supplied by the MCA (~2 mm posterior and ~5 mm lateral to bregma). Mice were randomly allocated to one of the treatment groups: i.p. twice with 0.2 ml of either vehicle (10% DMSO; n = 12), AVE0991 (10 mg/kg; n = 6) or AVE0991 (20 mg/kg; n = 12) at the commencement of reperfusion and again after a further 4 h. Some animals were also pre-treated with A779 1 h prior to the stroke surgery (80 mg/kg; n = 12). After recovery from anesthesia, mice were returned to their individual home cage. After surgery, mice were monitored 2–3 times daily and health status recorded according to Monash Animal Ethics Guidelines.

### Neurological Assessment

Neurological deficit was evaluated in a blinded fashion using a five-point scoring system (0, no deficit; 1, failure to extend right paw; 2, circling to the right; 3, falling to the right; 4, unable to walk spontaneously) and also by hanging grip test, as described previously [2, 21, 22]. Briefly, mice were suspended from a wire 30 cm above soft padding by their forelimbs for up to 60 s. Average hanging time (i.e. latency to fall) of 3 trials with 5 min rest in between was recorded.

### Open Field and Parallel Rod Floor Test

Locomotor activity and motor coordination were assessed on a parallel rod floor apparatus using ANY-maze software coupled to an automated video-tracking system used previously in mouse models of Alzheimer’s disease [23], duplication syndrome [24] and ataxia [25, 26] (ANY-maze Software, Stoelting Co.; Wood Dale, IL, USA) [27]. An animal was placed in a 20 x 20 cm chamber with parallel metal rods spaced 8 mm apart and suspended 1 cm above a metal floor. Mice were allowed to freely roam around the chamber, and each time a paw slipped and touched the metal floor beneath the parallel rods, a foot slip was detected and recorded by the software. Locomotor activity was recorded by an overhead video camera and analysed by the ANY-maze software during the 5 min test period.

### Quantification of Infarct Volume

Cerebral infarct and edema volume were estimated as described previously [21]. Infarct volume was the primary outcome of this experiment. Mice were euthanised by isoflurane inhalation followed by decapitation. The brain was removed immediately and snap frozen in liquid nitrogen. Coronal sections (30 μm; separated by ~420 μm) were cut and then stained with 0.1% thionin to delineate the infarct using ImageJ software (NIH, Bethesda, USA). The investigator was blinded as to the treatment groups when performing the analysis.

### Data Analysis

Data are presented as mean ± standard error of the mean (S.E.M), except for neurological deficit scores (median). Statistical significance (P<0.05) was determined by one-way analysis of variance (ANOVA) with Dunnett’s post-hoc test (*in vitro*) or Bonferroni’s multiple comparison tests (*in vivo*) as appropriate, using GraphPad Prism version 6.

## Results

### Effect of AVE0991 on neuronal cell death during glucose deprivation *in vitro*

**Fig 1:**
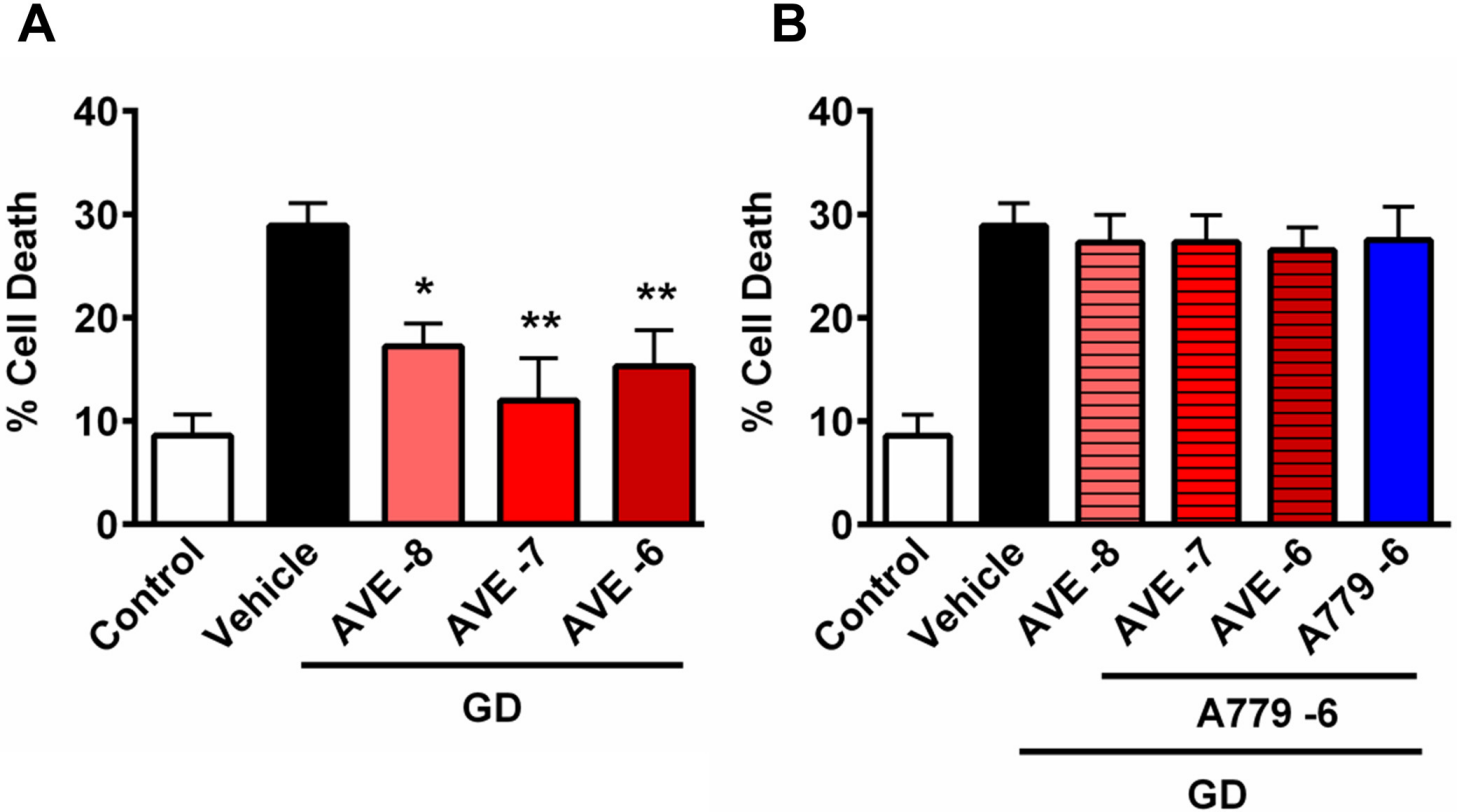
The effect of AVE0991 on neuronal cell death following glucose deprivation. Data are shown for cells exposed to normal conditions (control) or glucose deprivation (vehicle) for 24 h or with (A) AVE0991 or (B) AVE0991+A779. Data are presented as mean ± S.E.M (*P<0.05, **P<0.01 vs. vehicle; n = 6).

To investigate whether MasR stimulation may be neuroprotective, cultured primary cortical neurons were exposed to glucose deprivation for 24 h in the presence of the classical MasR agonist, AVE0991. When cells were deprived of glucose for 24 h, there was ~30% cell death, compared to ~8% death in control cells (Fig 1). Treatment with AVE0991 resulted in reduced neuronal death during glucose deprivation, and at 1x10^-7^ M reached near-complete protection of neurons in comparison to control cells (Fig 1A; P<0.01). Likewise, when AVE0991 (1x10^-7^ M) was applied 1 h after the onset of GD the protection was maintained, whereas the protective effect was lost if application occurred at later time points (i.e., 4 h and 8 h after GD) (S1 Fig). Moreover, neuroprotection was prevented when cells were co-incubated with AVE0991 and the MasR antagonist, A779 (Fig 1B). Note, A779 had no effect on neuronal cell death when administered to glucose-deprived cells alone.

### Effect of AVE0991 on outcomes following ischemic stroke

**Fig 2:**
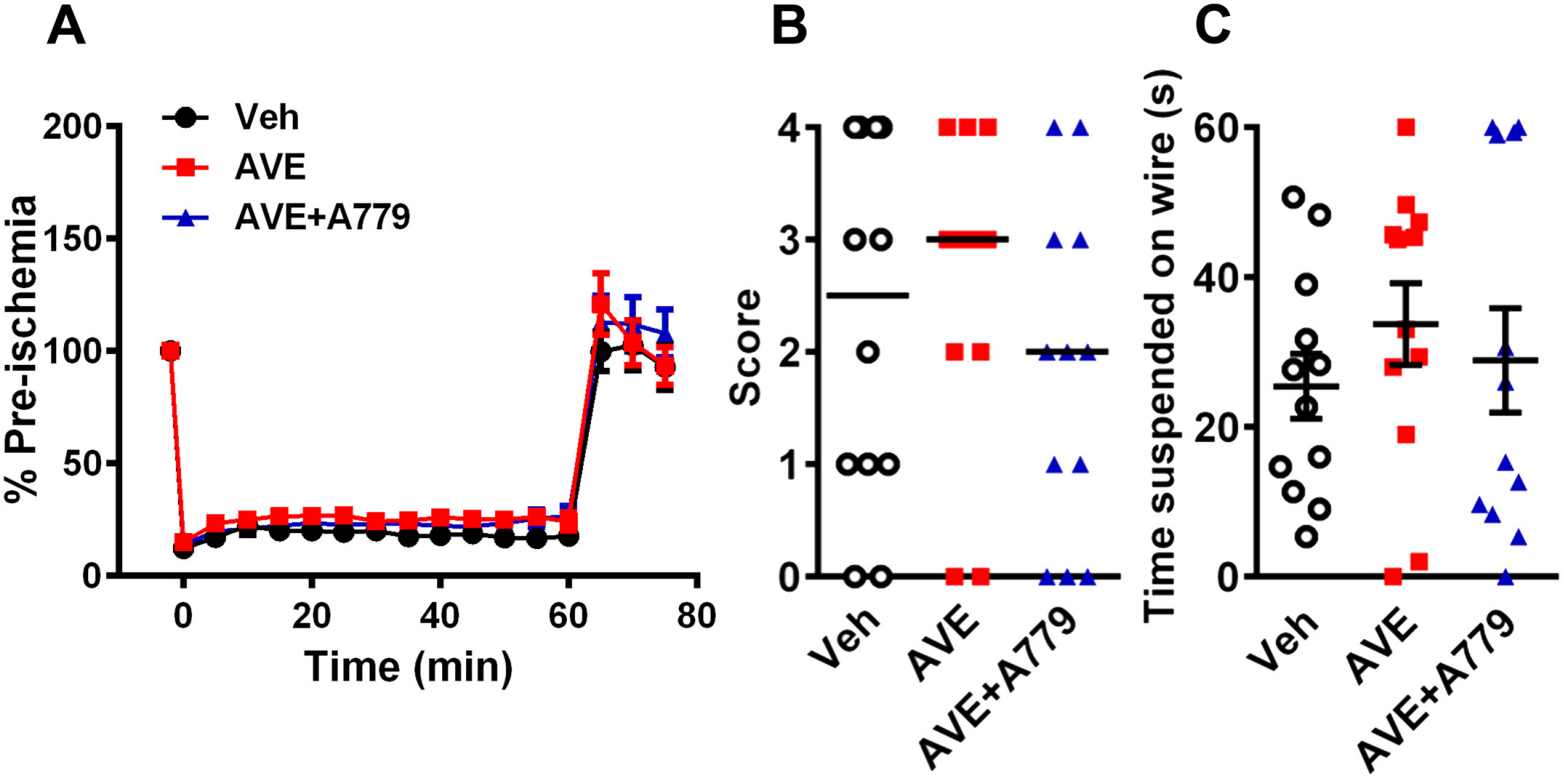
Regional cerebral blood flow and neurological function. Regional cerebral blood flow was recorded during and after 60 min MCAo with reperfusion. Data for (A) regional cerebral blood flow (n = 9–12), (B) neurological deficit (vehicle, n = 12; AVE0991, n = 12; AVE0991+A779, n = 12) and (C) hanging wire (vehicle, n = 12; AVE0991, n = 12; AVE0991+A779, n = 12) at 24 h post-MCAo. Data (A) and (C) are presented as mean ± S.E.M. Lines in (B) indicate median scores.

To evaluate the effect of MasR activation in ischemic stroke *in vivo*, mice were systemically administered vehicle or AVE0991 at the time of reperfusion. The regional cerebral blood flow profile did not differ between vehicle- and AVE-treated mice during 60 min of MCAo and for at least the first 15 min of reperfusion (Fig 2A; n = 12). Mice pretreated with AVE0991+A779 also showed a similar average blood flow profile, although AVE0991 administration at the beginning of reperfusion appeared to increase flow in some mice (Fig 2A; n = 12).

**Fig 3:**
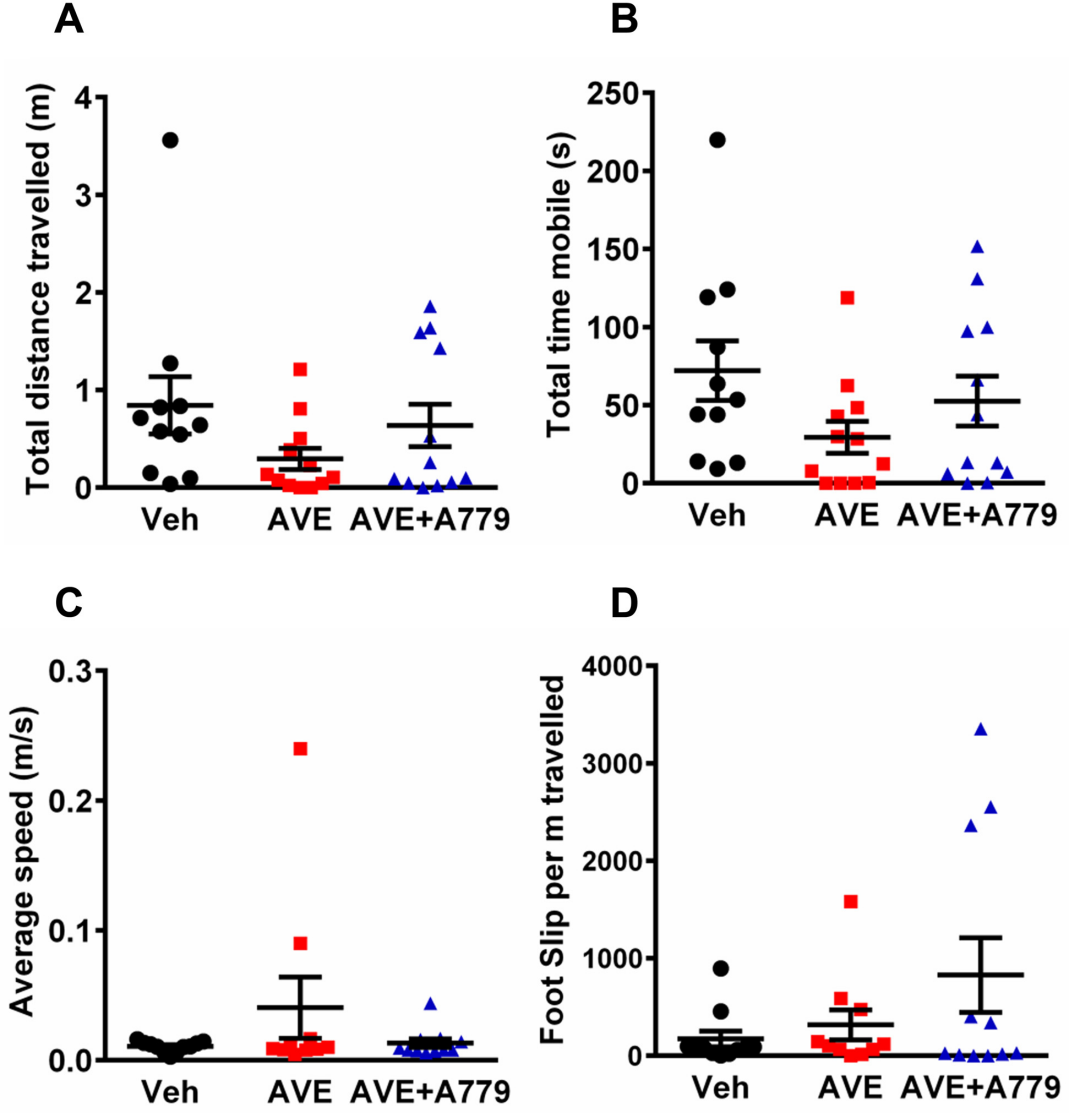
Locomotor activity and motor coordination test. Data for (A) total distance travelled, (B) total time mobile, (C) average speed while mobile and (D) foot slips per m travelled. Data are presented as mean ± S.E.M (n = 11–12).

**Fig 4:**
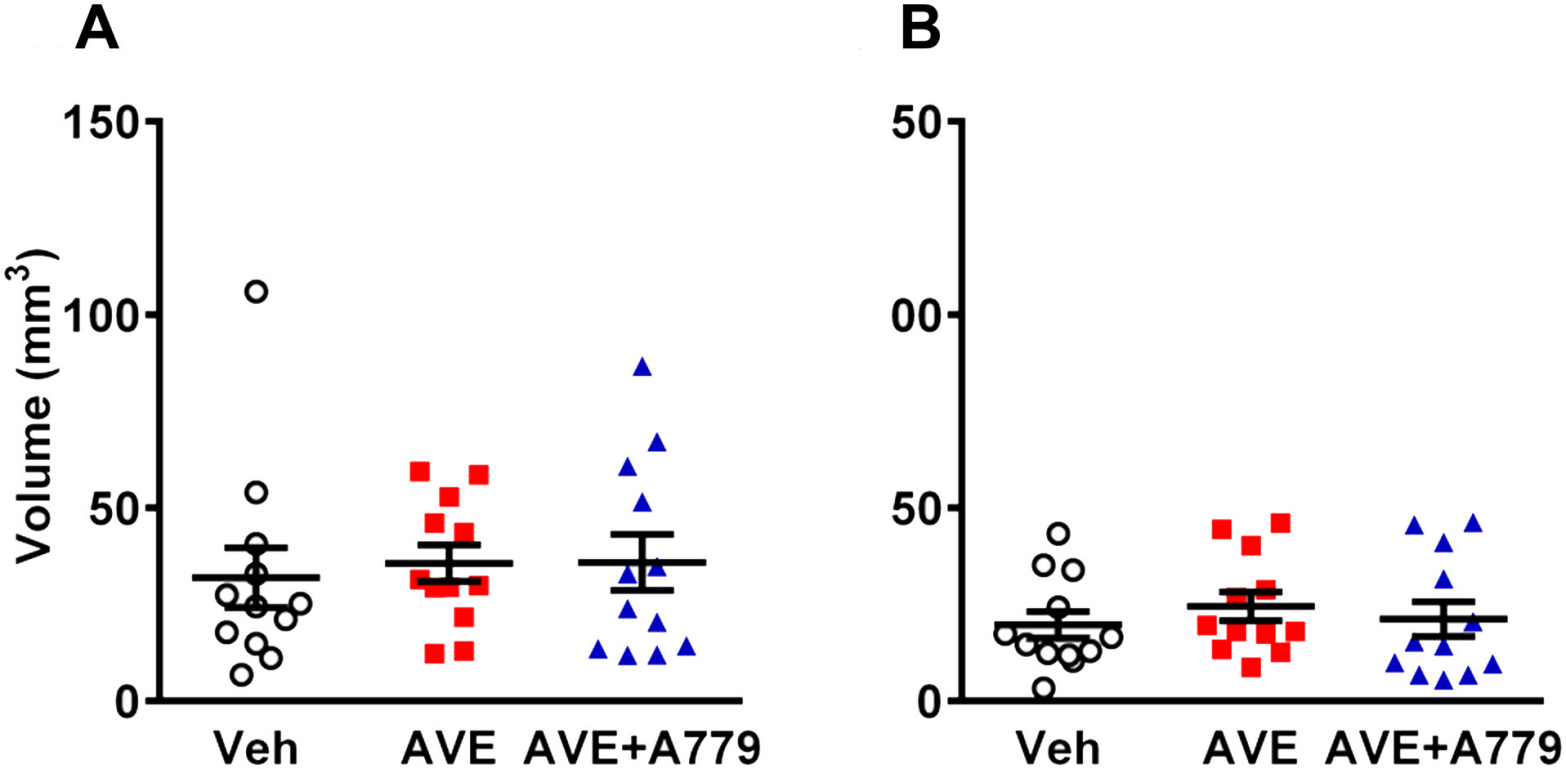
Cerebral infarct and edema volumes. Infarct volumes taken 24 h post-transient middle cerebral artery occlusion in (A) total infarct and (B) edema volumes (vehicle, n = 12; AVE0991, n = 12; AVE0991+A779, n = 12). Data are presented as mean ± S.E.M.

Mice treated with AVE0991 exhibited similar levels of neurological impairment to vehicle-treated mice after cerebral ischemia (Fig 2B; median neurological deficit score: veh, 2.5 vs AVE0991, 3; n = 12). In the hanging wire test, there was no apparent effect of AVE0991 treatment (Fig 2C; n = 12). Locomotor activity data indicated a trend for greater impairment in AVE0991-treated mice after stroke with total distance travelled and total time mobile both reduced by >50% compared with vehicle-treated mice, although these differences did not reach statistical significance (Fig 3A and 3B; n = 11–12). The average speed of the animals while mobile was similar in all 3 groups (Fig 3C; n = 11–12). In addition, mice treated with AVE0991 made similar numbers of foot slips compared to vehicle-treated mice, when corrected for the distance travelled (Fig 3D; n = 11–12). A779 treatment in combination with AVE0991 had no significant effect on any of the functional outcome measures (Figs 2B, 3A and 3B). Consistent with these behavioural/functional data, infarct and edema volumes were also similar in all three groups (Fig 4A and 4B; all n = 12). An intermediate dose of AVE0991 (10 mg/kg) also did not affect stroke outcome (S2, S3 and S4 Figs).

## Discussion

There is increasing interest in the MasR as an important cellular modulator and pharmacological target in studies of cardiovascular disease in general, including cerebrovascular conditions [14, 16, 28–31]. However, to date there is limited information on its role in the setting of stroke. In particular, the receptor(s) mediating the protective effects in cerebral ischemia of Ang (1–7), an endogenous agonist of both MasR and AT_2_R, have not yet been distinguished. Thus, this study has examined the efficacy of pharmacological MasR activation using its classical selective agonist, AVE0991. Experiments were performed both in primary neurons subjected to stroke-like conditions *in vitro* and in a mouse model of cerebral ischemia *in vivo*.

The results demonstrate that AVE0991 strongly protects neurons from ischemic injury *in vitro*, when applied early during the ischemic period. We confirmed that this neuroprotective effect of AVE0991 occurred in a MasR-dependent manner because it was blocked by the MasR antagonist, A779. Despite its clear neuroprotective effects in neuronal cells *in vitro*, systemic administration of AVE0991 had no such beneficial effects in our *in vivo* model of stroke, and to some extent it tended to worsen functional outcomes at 24 h (neurological deficit; locomotor activity).

Our finding that AVE0991 increased neuronal survival following glucose-deprivation in a MasR-dependent manner, is novel. Analogous to the neuroprotective effect of AVE0991 *in vitro* observed here, we previously found that direct stimulation of AT_2_R using CGP42112 can also protect cultured neurons exposed to glucose-deprivation. Moreover, systemic administration of CGP42112 improved functional outcome and reduced infarct volume in the same model of stroke as was used here [2].

Previous studies have reported that Ang (1–7) reduced infarct size, prevented the loss of neurons, and improved neurological outcome in rats subjected to endothelin-1-induced MCAo [14]. In addition, Jiang et al. (2012) have similarly reported that direct, intracerebral pretreatment with Ang (1–7) resulted in A779-sensitive protection in a permanent MCAo rat model of stroke [30]. Accordingly, we had predicted a similar improvement by the Ang (1–7) analogue and selective MasR agonist, AVE0991, following systemic administration shortly after ischemia-reperfusion in our mouse model of stroke. However, despite its positive *in vitro* effects on primary neurons, our *in vivo* data suggest that the systemic administration of a selective MasR agonist does not provide protection following stroke. Thus, it seems likely that the systemic route of administration is greatly inferior to local injection of AVE0991 in terms of limiting cerebral post-ischemic injury.

Previous studies have reported that AVE0991 crosses the blood-brain barrier [32, 33]. It is unlikely that the failure to achieve neuroprotection *in vivo* was due to an inadequate (or possibly excessive) dose of AVE0991 (10 or 20 mg/kg; i.e. 5 or 10 mg/kg administered at reperfusion and again 4 h post-stroke). Nonetheless, similar doses of AVE0991 (9 mg/kg and 15 mg/kg) have been reported to be protective in other models of disease, such as arthritis and renal ischemia-reperfusion [34, 35]. In pilot experiments, we found that a 1 mg/kg dose of AVE0991 also provided no neuroprotection *in vivo* (data not shown). Another potential limitation of the *in vivo* part of our study may have been the timing of AVE0991 administration, i.e. after initiating post-ischemic reperfusion, as opposed to an earlier administration (i.e. pre-treatment) that might be more effective. However, our interest here was to evaluate potential clinical utility of the drug by simulating post-stroke treatment. Furthermore, the potential for non-specific activation of MasR on other cell types, including astrocytes and endothelial cells, should not be overlooked as a possible confounding factor. Nevertheless, this is the first study to have tested post-treatment of AVE0991 in stroke and our data do not support the hypothesis that the MasR is a viable target for therapy in acute stroke.

In conclusion, this study has found that the classical MasR agonist, AVE0991, can directly and powerfully protect neurons from ischemia-like injury *in vitro*. However, such protective effects do not appear to translate to an *in vivo* stroke model in which the compound is administered systemically early after ischemia.

## Supporting Information

S1 Fig The effect of AVE0991 (1x10^-7^ M) applied after glucose deprivation. (DOCX)

S2 Fig Regional cerebral blood flow and neurological function. (DOCX)

S3 Fig Locomotor activity and motor coordination test. (DOCX)

S4 Fig Cerebral infarct and edema volumes. (DOCX)
