# Supplementary material for: Effect of a Selective Mas Receptor Agonist in Cerebral Ischemia In Vitro and In Vivo
Source: PLoS One. 2015 Nov 5;10(11):e0142087. doi: 10.1371/journal.pone.0142087 (PMC4634944; doi:10.1371/journal.pone.0142087)
Supplement: S1 Fig — (DOCX) [file pone.0142087.s001.docx]

**
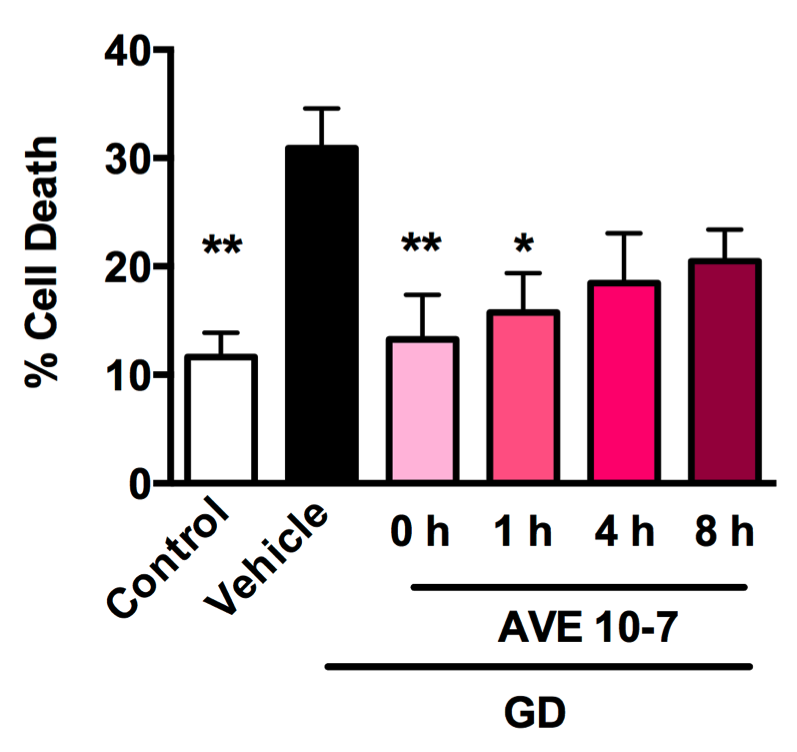
**

**S1 Fig: The effect of AVE0991 (1x10^-7^ M) applied after glucose deprivation.** Data are shown for cells exposed to normal conditions (control) or glucose deprivation (vehicle) for 24 h or with AVE0991 (1x10^-7^ M) administered at 0 h, 1 h, 4 h and 8 h following glucose deprivation. Data are presented as mean ± S.E.M (**P*<0.05, ***P*<0.01 vs. vehicle; n=6).
