# Supplementary material for: Effect of a Selective Mas Receptor Agonist in Cerebral Ischemia In Vitro and In Vivo
Source: PLoS One. 2015 Nov 5;10(11):e0142087. doi: 10.1371/journal.pone.0142087 (PMC4634944; doi:10.1371/journal.pone.0142087)
Supplement: S2 Fig — (DOCX) [file pone.0142087.s002.docx]

**
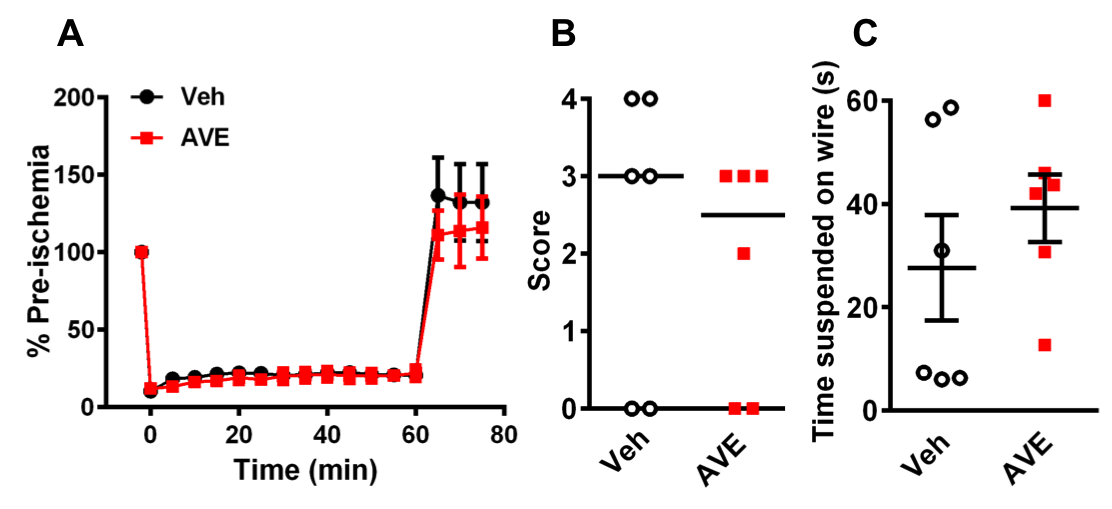
**

**S2 Fig: Regional cerebral blood flow and neurological function.** Regional cerebral blood flow was recorded during and after 60 min MCAo with reperfusion. Data for (A) regional cerebral blood flow (n=6), (B) neurological deficit (vehicle, AVE0991 (10 mg/kg); n=6) and (C) hanging wire (vehicle, AVE0991 (10 mg/kg); n=6) at 24 h post-MCAo. Data (A) and (C) are presented as mean ± S.E.M. Lines in (B) indicate median scores.
