# Supplementary material for: Effect of a Selective Mas Receptor Agonist in Cerebral Ischemia In Vitro and In Vivo
Source: PLoS One. 2015 Nov 5;10(11):e0142087. doi: 10.1371/journal.pone.0142087 (PMC4634944; doi:10.1371/journal.pone.0142087)
Supplement: S3 Fig — (DOCX) [file pone.0142087.s003.docx]

**
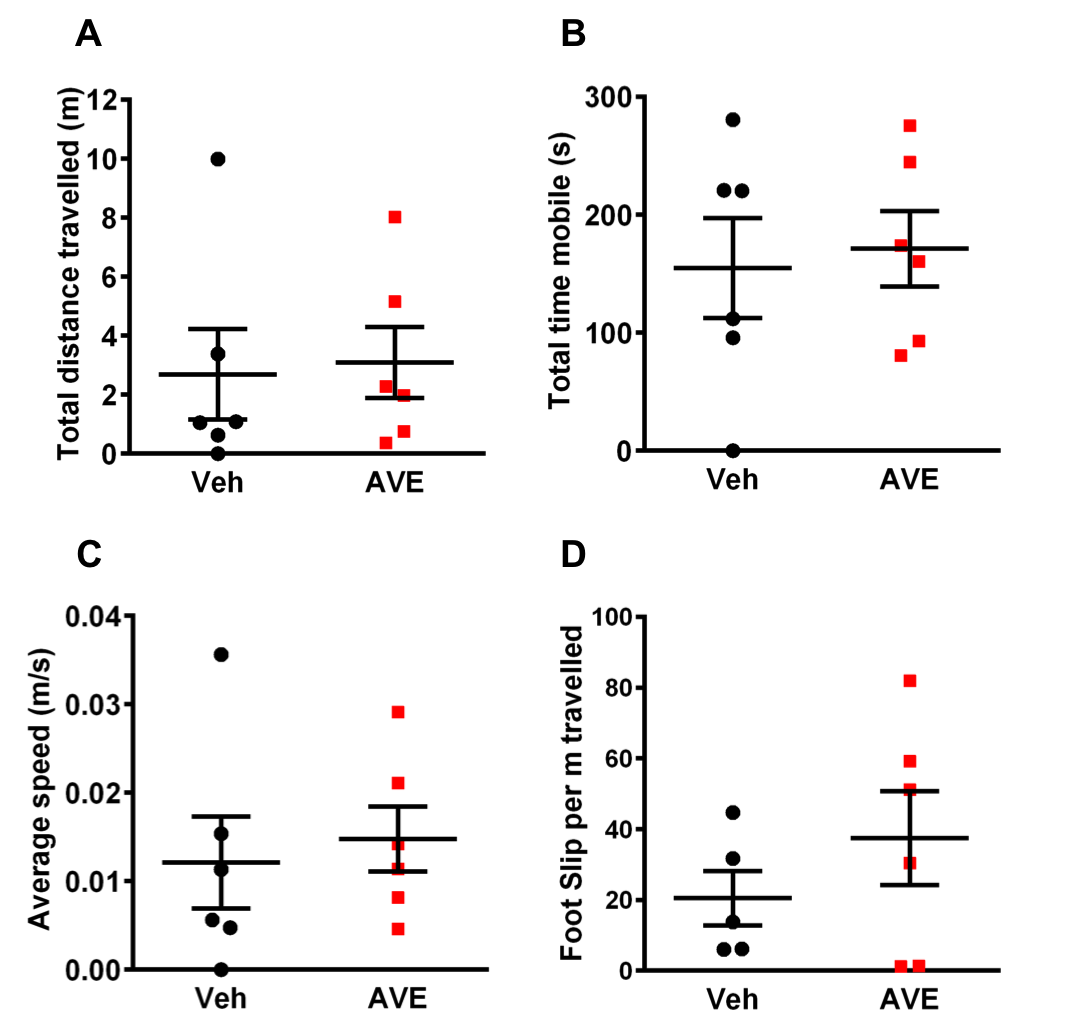
**

**S3 Fig: Locomotor activity and motor coordination test.** Data for (A) total distance travelled, (B) total time mobile, (C) average speed while mobile and (D) foot slips per m travelled. Data are presented as mean ± S.E.M (n=6).
