# Supplementary material for: Effect of a Selective Mas Receptor Agonist in Cerebral Ischemia In Vitro and In Vivo
Source: PLoS One. 2015 Nov 5;10(11):e0142087. doi: 10.1371/journal.pone.0142087 (PMC4634944; doi:10.1371/journal.pone.0142087)
Supplement: S4 Fig — (DOCX) [file pone.0142087.s004.docx]

**
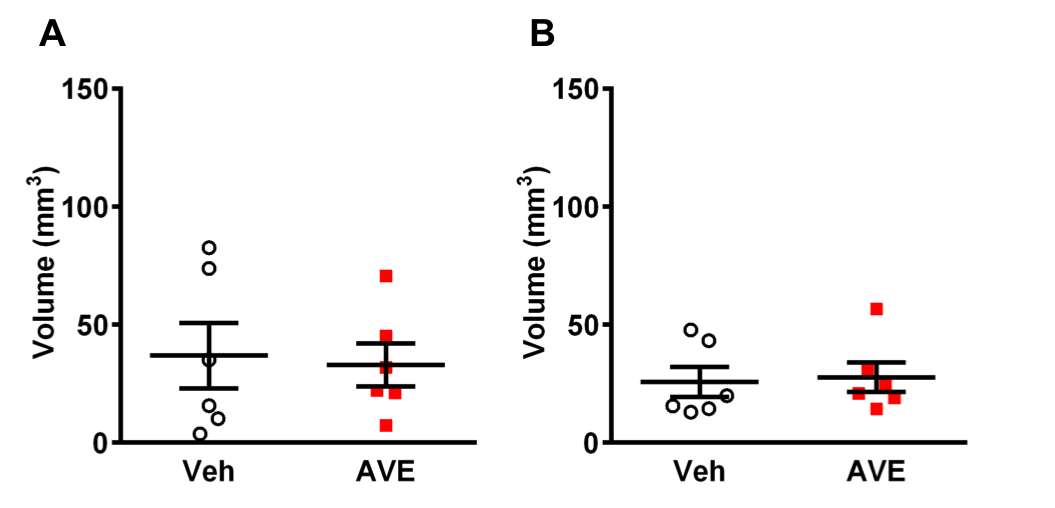
**

**S4 Fig:** **Cerebral infarct and edema volumes.** Brain injury at 24 h post-transient middle cerebral artery occlusion, including (A) total infarct volume and (B) edema volume (vehicle, AVE0991 (10 mg/kg); n=6). Data are presented as mean ± S.E.M.
